# A novel LASSO‐derived prognostic model predicting survival for non‐small cell lung cancer patients with M1a diseases

**DOI:** 10.1002/cam4.4560

**Published:** 2022-02-06

**Authors:** Hongchao Chen, Chen Huang, Huiqing Ge, Qianshun Chen, Jing Chen, Yuqiang Li, Haiyong Chen, Shiyin Luo, Lilan Zhao, Xunyu Xu

**Affiliations:** ^1^ Department of Thoracic Surgery, Fujian Provincial Hospital Shengli Clinical College of Fujian Medical University Fuzhou Fujian China; ^2^ Department of Pharmacy Fujian Children's hospital Fuzhou Fujian China; ^3^ Department of General Surgery, Xiangya Hospital Central South University Changsha Hunan China

**Keywords:** nomogram, non‐small cell lung cancer, prognostic, SEER

## Abstract

**Introduction:**

The current American Joint Committee on Cancer (AJCC) M1a staging of non‐small cell lung cancer (NSCLC) encompasses a wide disease spectrum, showing diverse prognosis.

**Methods:**

Patients who diagnosed in an earlier period formed the training cohort, and those who diagnosed thereafter formed the validation cohort. Kaplan–Meier analysis was performed for the training cohort by dividing the M1a stage into three subgroups: (I) malignant pleural effusion (MPE) or malignant pericardial effusion (MPCE); (II) separate tumor nodules in contralateral lung (STCL); and (III) pleural tumor nodules on the ipsilateral lung (PTIL). Gender, age, histologic, N stage, grade, surgery for primary site, lymphadenectomy, M1a groups, and chemotherapy were selected as independent prognostic factors using the least absolute shrinkage and selection operator (LASSO) Cox regression analysis. And a nomogram was constructed using Cox hazard regression analysis. Accuracy and clinical practicability were separately tested by Harrell's concordance index, the receiver operating characteristic (ROC) curve, calibration plots, residual plot, the integrated discrimination improvement (IDI), net reclassification improvement (NRI), and decision curve analysis (DCA).

**Results:**

The concordance index (0.661 for the training cohort and 0.688 for the validation cohort) and the area under the ROC curve (training cohort: 0.709 for 1‐year and 0.727 for 2‐year OS prediction; validation cohort: 0.737 for 1‐year and 0.734 for 2‐year OS prediction) indicated satisfactory discriminative ability of the nomogram. Calibration curve and DCA presented great prognostic accuracy, and clinical applicability. Its prognostic accuracy preceded the AJCC staging with evaluated NRI (1‐year: 0.327; 2‐year: 0.302) and IDI (1‐year: 0.138; 2‐year: 0.130).

**Conclusion:**

Our study established a nomogram for the prediction of 1‐ and 2‐year OS in patients with NSCLC diagnosed with stage M1a, facilitating healthcare workers to accurately evaluate the individual survival of M1a NSCLC patients. The accuracy and clinical applicability of this nomogram were validated.

## Graphical abstract

The newly defined M1a subgroups showed diverse prognostic performance. MPE and MPCE were significant factors for poorer OS. The prognostic model could effectively predict 1‐ and 2‐year OS.

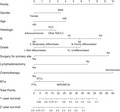

## 1 INTRODUCTION

Lung cancer, a prevalent malignancy, is the leading cause of global cancer‐associated mortalities.^1^ The main pathological types of lung cancer are classified as non‐small cell lung cancer (NSCLC) as well as small‐cell lung cancer (SCLC), accounting for 85% and 15% of all lung cancer, respectively.^2^ At initial diagnosis, many NSCLC patients are characterized by malignant pleural effusion (MPE) or malignant pericardial effusion (MPCE).^3^ These patients are categorized as M1a stage based on the seventh edition American Joint Committee on Cancer (AJCC) tumor‐node‐metastasis (TNM) staging system. Besides MPE and MPCE, separate tumor nodules in contralateral lung (STCL) and pleural tumor nodules on ipsilateral lungs (PTIL) are also included in M1a.^4^ Interestingly, several studies found diverse prognosis among different metastatic sites in M1a patients,^5^ with median overall survival ranging from 3–8 months,^6^, ^7^ suggesting a high heterogeneity within the M1a stage.

The existing AJCC staging system is only dependent on tumor size (T), the presence or absence of nodal status (N), and metastasis (M), lacking an evaluation of clinicopathologic characteristics including age, gender, histology, metastatic organ, the number of metastatic sites, as well as modality of treatment,^8^ which could also affect the prognosis. It is obvious that the TNM system is not sufficient for predicting outcome in an individual patient. Accurate risk stratification allows patients and physicians to better balance pros and cons while making decisions.^9^ Therefore, a more accurate and comprehensive tool is needed.

Nomogram, a visual risk regression model, is an ideal tool for the prediction of patients' prognostic outcomes.^10^ Various nomograms have been constructed for the prognostic prediction of metastatic NSCLC patients, such as with distant organ metastasis,^11^ with MPE or MPCE.^12^ In 2020, there was a study created a nomogram focus on M1a NSCLC patients.^13^ Several clinical characteristics and clinicopathological variables were included in the nomogram. Nevertheless, the metastatic site, which has been demonstrated to influence overall survival in M1a NSCLC patients^14^ and should be considered for predicting individualized prognosis, was not included as a predictor. Thus, there still absent efficient model to predict the survival of NSCLC patients with various M1a descriptor. Herein, we aimed at establishing a novel nomogram to assess relevant risk factors and estimate overall survival and provide a satisfying prognostic indication to NSCLC patients initially diagnosed with different metastatic sites in the M1a stage.

## 2 METHODS

### 2.1 Study population and data processing

SEER*stat software (version 8.3.5) was used to retrieve cases from the Surveillance, Epidemiology, and End Results (SEER) database, which is a public national database that covers about 28% of the US population.^15^ Data were collected only after official permission had been grated (username: 16695‐nov2019). The inclusion criteria were: (I) lung cancer was diagnosed pathologically from 2010 to 2015 (Site codes: C34.0, C34.1, C34.2, C34.3, C34.8, and C34.9); (II) pathologically confirmed NSCLC, the common histological types were included as follows: adenocarcinoma (ADC; 8140, 8141, 8230, 8244, 8245, 8250–8255, 8260, 8290, 8310, 8320, 8323, 8333, 8410, 8470, 8480, 8481, 8490, 8507, 8550, 8551, 8570, 8571, 8574, 8576), squamous cell carcinoma (SCC; 8052, 8070–8075, 8078, 8083, 8084, 8123), and other non‐small cell carcinoma (other NSCLC; 8004, 8012–8014, 8022, 8030–8035, 8046, 8082, 8200, 8240, 8249, 8430, 8560, 8562); (III) lung cancer as the first and only primary cancer diagnosis; and (IV) the patient's initial diagnosis was accompanied by one of malignant pleural effusion (MPE) or malignant pericardial effusion (MCPE) or separate tumor nodules in contralateral lung (STCL) or pleural tumor nodules on the ipsilateral lung (PTIL) (CS Mets at dx code: 15–18, MPE; 20, MPCE; 23, STCL; and 24, PTIL). Patients were excluded if: (I) their survival time was either 0 month or unknown; or (II) patients with absent or incomplete data regarding race, marital status, primary site, laterality, T stage, N stage, grade, surgery, and radiation.

Patients' clinical characteristics (age, gender, and marital status), clinicopathological (histology, laterality, primary site, seventh edition AJCC system T and N stage, M1a group, surgery for primary site, survival time [months], lymphadenectomy, surgical resection of metastatic lesions, radiation, and chemotherapy), and vital status recode were collected from the SEER database. Age as a continuous variable was separated into two groups (< 60 years and ≥ 60 years). Treatment with surgery and radiotherapy were separated into two categories (“No” or “Yes”), while chemotherapy was separated into “No/Unknown” or “Yes”. Overall survival (OS) was defined as the time between the date of diagnosis and death of any cause or the last follow‐up. OS was chosen as the primary outcome. The survival difference among each descriptor in the M1a stage was evaluated using Log‐rank test. Depending on the results of the Log‐rank test, we then reclassified M1a into three subgroups (MPE/MPCE, STCL, and PTIL).

### 2.2 Statistical analysis

To develop the nomogram and for further external validation, eligible patients who diagnosed between 2010 and 2013, were included into the training cohort, and those between 2014 and 2015 were entered into the validation cohort.^16^, ^17^ Descriptive analyses of demographic as well as clinicopathological characteristics of included study patients in the training as well as validation cohorts, and the median survival time with a 95% confidence interval (CI) for each subgroup was calculated using the Kaplan–Meier survival analysis. Categorical variables were compared using the chi‐squared test.

As least absolute shrink and selection operator (LASSO) Cox regression can effectively avoid redundancy or overfitting that occurs in significant feature selection,^18^, ^19^ we used this regression model in a training cohort to identify independent risk factors that affect OS. Along with an increase of a penalty factor (λ), the coefficients of the respective variables decrease. When the λ is the optimal, the coefficients of some variables are compressed to 0, at which point the variables that retains non‐zero are the final selected variable. Fivefold cross‐validation was used to determine optimal LASSO penalty. The resulting variables were included into the nomogram. The nomogram adopted a 1‐ and 2‐year OS as the endpoint. To determine whether the nomogram could distinguish between patients exhibiting dissimilar outcomes, discrimination for internal validation in the training cohort as well as external validation in the validation set was evaluated using Harrell's concordance index (C‐index) with a 95% CI, the receiver operating characteristic (ROC) curve and the area under the curve (AUC).^20^, ^21^ The C‐index is a value between 0.5 and 1, with 0.5 indicating that the model is completely random and 1 indicating that the model has perfect predictivity. We developed calibration plots for both the training and validation cohorts according to a fivefold cross‐validation and 1,000 bootstrap resamples to establish the concordance between predicted as well as observed 1‐ and 2‐year OS outcomes to assess the nomogram's predictive accuracy. The goodness‐of‐fit of the LASSO Cox regression model was illustrated by the Cox‐Snell residual plot.^22^ The model's reliability was examined using decision curve analysis (DCA), which has unique advantages in assessing the clinical benefit and utility of nomograms.^23^ Comparison of the nomogram and 7th edition AJCC TNM staging system was done with an integrated discrimination improvement (IDI) and net reclassification improvement (NRI).

Statistical analyses were conducted by SPSS 26.0 (SPSS Inc., Chicago, IL) or R software (version 3.6.1; http://www.r‐project.org/). All *P* values were two‐side, *P* < 0.05 was considered statistically significant.

## 3 RESULTS

### 3.1 Baseline clinicopathological features

**FIGURE 1:**
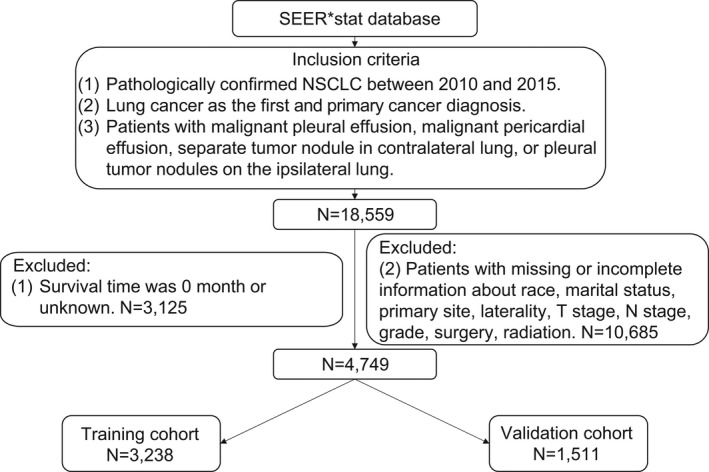
Schematic presentation of case selection. SEER: Surveillance, epidemiology, and end results. NSCLC: Non‐small cell lung cancer

**FIGURE 2:**
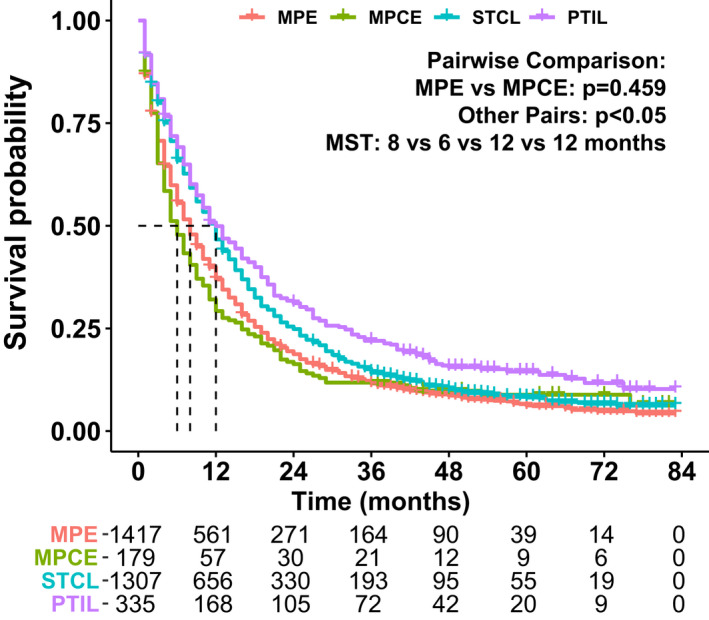
Kaplan–Meier survival curves for overall survival stratified by metastatic pattern. MPE: Malignant pleural effusion; MPCE: Malignant pericardial effusion; STCL: Separate tumor nodules in contralateral lung; PTIL: Pleural tumor nodules on the ipsilateral lung; MST: Median survival time

We finally collected a total of 4,749 cases. A specific screening flowchart is shown in Figure 1. The whole cohort was entered into two groups, where 3,238 and 1,511 cases were included in the training and validation cohorts, respectively. Demographic as well as clinicopathological characteristics of patients and their OS (95% CI) are presented in Table 1. In the training cohort, ADC accounted for the highest proportion, with 1,689 (52.16%) patients having ADC, whereas those with squamous cell carcinoma and other non‐small cell carcinoma were 1,181 (36.47%) and 368 (11.37%), respectively. A total of 1,920 (59.30%) patients had chemotherapy and 1,318 (40.70%) patients had no chemotherapy or chemotherapy status was unknown. The 1‐year survival rate was 36.9% for MPE, 29.2% for MPCE, 46.7% for STCL, and 49.9% for PTIL. The 2‐year survival rate for MPE, MPCE, STCL, and PTIL was 18.7%, 16.3%, 24.8%, and 31.2%, respectively. As shown in Figure 2, statistically significant differences were found among each M1a descriptor except for MPE and MPCE (*P* = 0.459). The M1a stage was then divided into three subgroups: (I) MPE or MPCE; (II) STCL; and (III) PTIL. There were 1,596 (49.29%) patients with MPE/MPCE, 1,307 (40.36%) patients with STCL, and 335 (10.35%) patients with PTIL.

**TABLE 1:** Demographic and clinicopathological characteristics of the training cohort and validation cohort

Demographic or clinicopathological characteristics	Training set (N = 3,238)			Validation set (N = 1,511)			*P* value^a^
	No. of patients (%)	OS (months)		No. of patients (%)	OS (months)		
		Median	95% CI		Median	95% CI	
Gender							0.189
Male	1,787 (55.19)	8	7.338–8.662	803 (53.14)	9	7.874–10.126	
Female	1,451 (44.81)	11	10.033–11.967	708 (46.86)	12	10.496–13.504	
Race							0.253
White	2,476 (76.47)	9	8.361–9.639	1,122 (74.26)	10	8.919–11.081	
Black	469 (14.48)	10	8.238–11.762	240 (15.88)	10	8.051–11.949	
Others	293 (9.05)	13	10.338–15.662	149 (9.86)	14	8.946–19.054	
Age							0.331
< 60	661 (20.41)	13	11.527–14.473	290 (19.19)	15	12.047–17.953	
≥ 60	2,577 (79.59)	9	8.438–9.562	1,221 (80.81)	9	8.061–9.939	
Marital status							0.366
Married	1,649 (50.93)	11	10.144–11.856	748 (49.50)	12	10.543–13.457	
Unmarried	1,589 (49.07)	9	8.246–9.754	763 (50.50)	9	7.814–10.186	
Primary site							0.796
Main bronchus	195 (6.02)	6	4.643–7.357	81 (5.36)	6	3.648–8.352	
Upper lobe	1,822 (56.27)	10	8.229–10.771	848 (56.12)	11	9.829–12.171	
Middle lobe	169 (5.22)	10	8.210–11.790	78 (5.16)	10	5.028–14.972	
Lower lobe	1,052 (32.49)	9	8.009–9.991	504 (33.36)	11	9.336–12.664	
Laterality							0.395
Left	1,393 (43.02)	10	9.053–10.947	630 (41.69)	11	9.248–12.752	
Right	1,845 (56.98)	9	8.312–9.688	881 (58.31)	10	8.892–11.108	
Histologic type							0.001
ADC	1,689 (52.16)	12	11.025–12.975	859 (56.85)	14	12.385–15.615	
SCC	1,181 (36.47)	8	7.189–8.811	527 (34.88)	8	7.132–8.868	
Other NSCLC	368 (11.37)	7	5.852–8.148	125 (8.27)	6	3.923–8.077	
T stage							0.317
T1	210 (6.49)	16	12.633–19.367	84 (5.56)	18	8.376–27.624	
T2	789 (24.37)	10	8.927–11.073	388 (25.68)	11	9.127–12.873	
T3	932 (28.78)	9	7.830–10.170	455 (30.11)	10	8.183–11.817	
T4	1,307 (40.36)	9	8.102–9.898	584 (38.65)	9	7.811–10.189	
N stage							0.072
N0	1,015 (31.35)	12	10.606–13.394	524 (34.65)	14	11.812–16.188	
N1	265 (8.18)	9	7.021–10.979	126 (8.34)	11	7.969–14.031	
N2	1,480 (45.71)	9	8.197–9.803	633 (41.89)	9	7.777–10.223	
N3	478 (14.76)	8	6.949–9.051	228 (15.09)	8	6.716–9.284	
M1a group							0.003
MPE/MPCE	1,596(49.29)	8	7.340–8.660	670 (44.34)	8	7.150–8.850	
STCL	1,307 (40.36)	12	11.103–12.897	651 (43.08)	12	10.275–13.725	
PTIL	335 (10.35)	12	9.515–14.485	190 (12.58)	17	12.713–21.287	
Grade							0.025
I, WD	251 (7.75)	15	12.671–17.329	148 (9.79)	20	16.041–23.959	
II, MD	1,114 (34.41)	12	11.000–13.000	550 (36.40)	13	11.110–14.890	
III, PD	1,803 (55.68)	8	7.301–8.699	781 (51.69)	8	7.137–8.863	
IV, UD	70 (2.16)	7	4.267–9.733	32 (2.12)	5	0.000–10.544	
Surgery for primary site							0.770
No	2,868 (88.57)	9	8.476–9.524	1,334 (88.29)	9	8.164–9.836	
Yes	370 (11.43)	25	20.650–29.350	177 (11.71)	23	16.535–29.465	
Lymphadenectomy							0.063
No	2,817 (87.00)	9	8.461–9.539	1,284 (84.98)	9	8.093–9.907	
Yes	421 (13.00)	19	16.025–21.975	227 (15.02)	20	14.490–25.510	
Surgery for metastasis site							0.330
No	3,165 (97.75)	9	8.424–9.576	1,484 (98.21)	10	9.070–10.930	
Yes	73 (2.25)	16	9.548–22.452	27 (1.79)	26	NA	
Lymph nodes biopsy							0.003
Positive	292 (9.02)	13	9.733–16.267	183 (12.11)	12	8.776–15.224	
Negative	190 (5.87)	25	19.211–30.789	94 (6.22)	NA	NA	
No	2,756 (85.11)	9	8.450–9.550	1,234 (81.67)	9	8.072–9.928	
Radiotherapy							0.040
Yes	1,179 (36.41)	10	9.175–10.825	504 (33.36)	11	9.810–12.190	
No	2,059 (63.59)	9	8.200–9.800	1007 (66.64)	10	8.792–11.208	
Chemotherapy							1.000
Yes	1,920 (59.30)	13	12.221–13.779	896 (59.30)	15	13.434–16.566	
No/Unknown	1,318 (40.70)	5	4.482–5.518	615 (40.70)	6	5.097–6.903	

Abbreviations: CI: confidence interval; ADC: adenocarcinoma; SCC: squamous cell carcinoma; NSCLC: non‐small cell lung cancer; MPE: malignant pleural effusion; MPCE: malignant pericardial effusion; STCL: separate tumor nodules in contralateral lung; PTIL: pleural tumor nodules on ipsilateral lung; WD: well differentiated; MD: moderately differentiated; PD: poorly differentiated; UD: undifferentiated; NA: not available.

^a^ Categorical variables among training and validation cohort were compared using the χ^2^ test.

### 3.2 Independent prognostic factors selection

**FIGURE 3:**
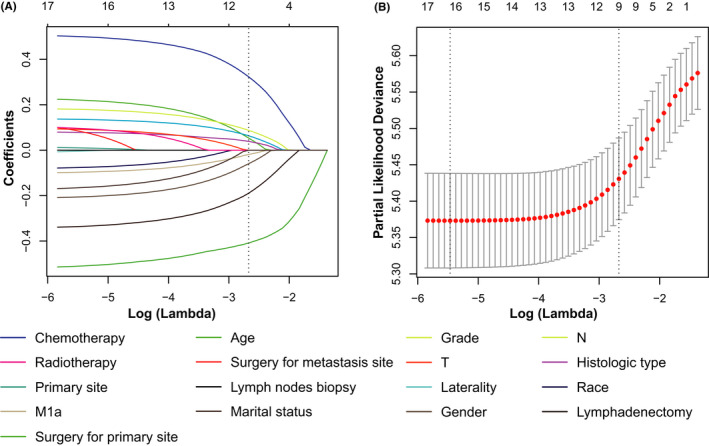
Feature selection using the least absolute shrinkage and selection operator (LASSO) COX regression. (**A**) Profiles of LASSO coefficient for clinical and pathological features. (**B**) Selection of tuning parameter (lambda) in the LASSO regression using fivefold cross validation

A total of 17 variables were included in the LASSO Cox regression. After LASSO Cox regression (Figure 3), nine variables with nonzero coefficients remained significant predictors of OS, including: gender, age, histology, grade, N stage, M1a stage, surgery for primary site, lymphadenectomy, and chemotherapy.

### 3.3 Nomogram establishment and validation

**FIGURE 4:**
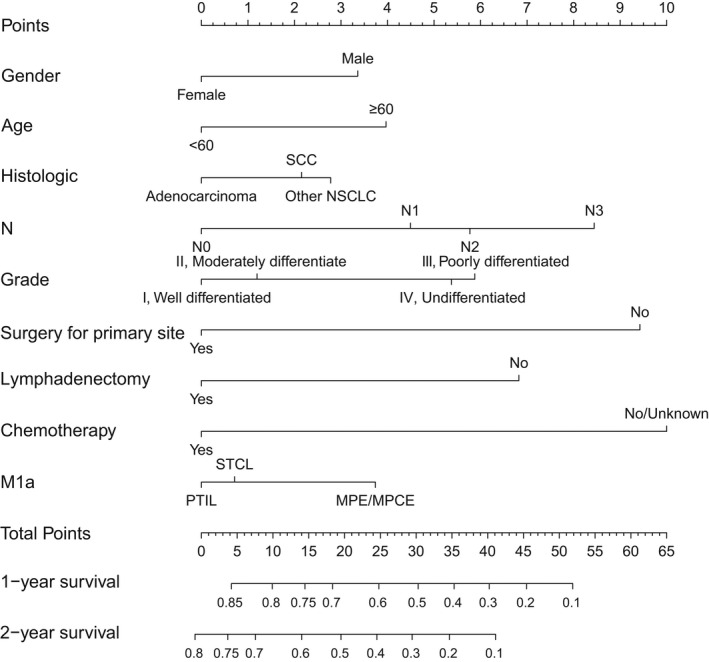
Prognostic nomogram for NSCLC patients with M1a disease. Points for each variable were added to establish total points after which final scores were used in estimation of 1‐ and 2‐year survival outcomes. NSCLC: Non‐small cell lung cancer; MPCE: Malignant pericardial effusion; STCL: Separate tumor nodules in contralateral lung; MPE: Malignant pleural effusion; PTIL: Pleural tumor nodules on the ipsilateral lung

**FIGURE 5:**
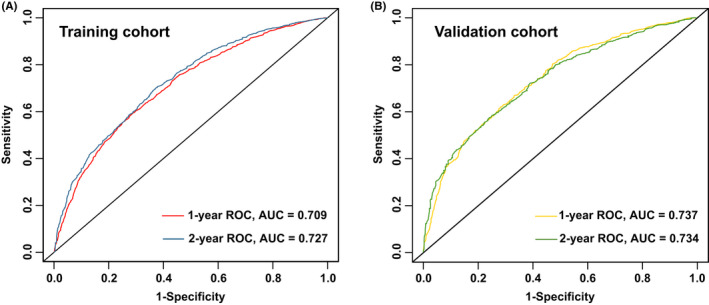
Receiver operating characteristic (ROC) curves were generated from the training (**a**) and validation (**B**) dataset to test the performance evaluating of the newly established nomogram, by the areas under the ROC curves (AUC)

**FIGURE 6:**
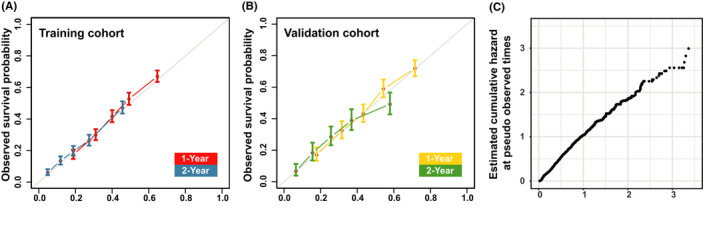
Calibration plots of 1‐ and 2‐year overall survival for the training cohort (**A**) and validation cohort (**B**). Observed as well as estimated survival outcomes are plotted on the y‐axis and x‐axis, respectively. Means of predicted survival outcomes from our model were compared to means of observed survival outcomes as determined by the Kaplan–Meier after grouping of equal sample sizes. The 45‐degree line through the origin point denotes a flawless calibration model with matching actual as well as estimated survival outcome possibilities. The vertical arrows denote 95% CIs for observed survival. Cox‐Snell residual to assess the fit of the LASSO‐Cox model (**C**), Cox‐Snell residuals as pseudo observed times as well as estimated cumulative hazard at pseudo observed times are plotted on the x‐axis and y‐axis, respectively. And the slope is approximately 45 degrees

A nomogram was constructed with a basis on the resulting nine variables, and each subgroup within these variables was allocated a score (Table 2). The points from the various variables were summed to obtain a total point, and the predicted 1‐ as well as 2‐year survival probabilities were obtained by plotting the vertical lines from the total point’s axis to the two outcome axes (Figure 4). C‐index of the nomogram was 0.661 (95% CI: 0.650–0.672) in the training cohort and 0.688 (95% CI: 0.671–0.704) in the validation cohort. In the training cohort, the AUC for 1‐year OS was 0.709 and for 2‐year was 0.727 (Figure 5(A)). And in the validation cohort, the AUC for 1‐ as well as 2‐year OS was 0.737, 0.734, respectively (Figure 5(B)). Calibration for 1‐ as well as 2‐year OS outcomes exhibited a satisfactory agreement between the estimated and actual survival outcomes in both the training and validation cohorts (Figure 6(A), (B)), the Cox‐Snell residual plot also showed a good fitness of our nomogram (Figure 6(C)). Therefore, the nomogram has considerable discriminative as well as calibration abilities.

**TABLE 2:** Score of every subgroup within each variable

Variable	Points
Gender	
Male	3
Female	0
Age	
<60	0
≥60	4
Histology	
Adenocarcinoma	0
Squamous cell carcinoma	2
Other NSCLC	3
N stage	
N0	0
N1	4
N2	6
N3	8
Grade	
I	0
II	1
III	6
IV	5
Surgery for primary site	
No	9
Yes	0
Lymphadenectomy	
No	7
Yes	0
M1a group	
MPE/MPCE	4
STCL	1
PTIL	0
Chemotherapy	
Yes	0
No/Unknown	10

Abbreviations: NSCLC: non‐small cell lung cancer; MPE: malignant pleural effusion; MPCE: malignant pericardial effusion; STCL: separate tumor nodules in contralateral lung; PTIL: pleural tumor nodules on ipsilateral lung.

### 3.4 Comparison of the nomogram & 7th edition AJCC TNM staging system

**FIGURE 7:**
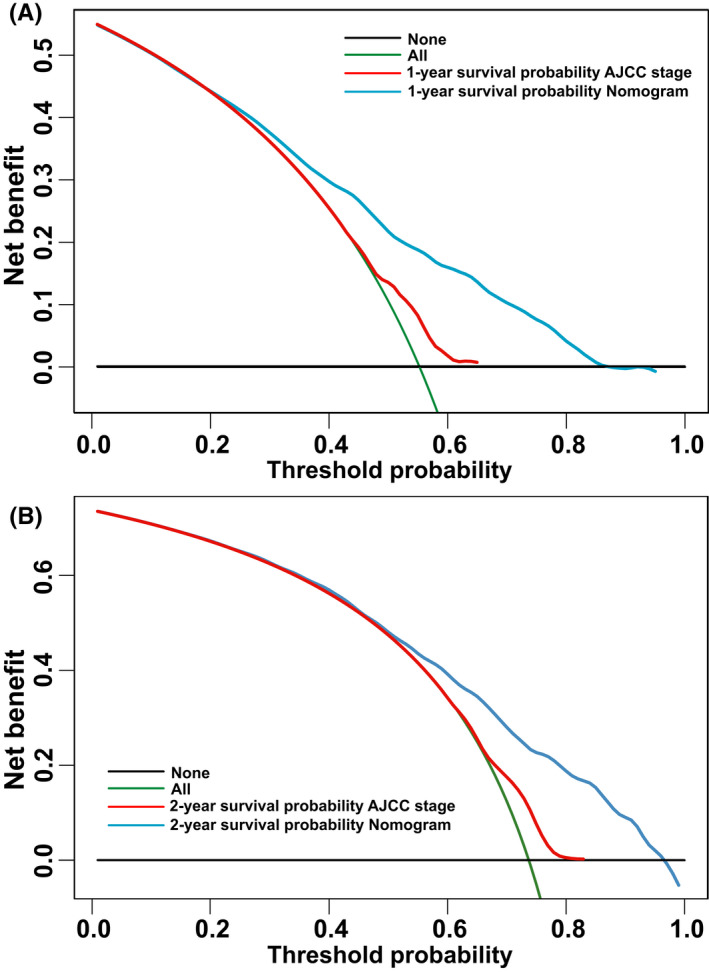
Decision curve analysis for the nomogram and AJCC stage in estimation of prognostic outcomes for M1a NSCLC patients at 1‐year (**A**) and 2‐years (**B**) in the validation cohort. The abscissa is the threshold probability, and the ordinate is the net benefit rate. Horizontal denotes that overall death occurred in no patients, with a net benefit of zero. Green shows all patients will have overall death at a specific threshold probability. AJCC: American joint committee on caner; NSCLC: Non‐small cell lung cancer

We performed DCA using a validation cohort to assess the clinical utility of our model and AJCC TNM staging. As shown in Figure 7, the DCA curves indicate that the nomogram has better clinical applicability in predicting 1‐ and 2‐year outcomes for M1a NSCLC patients. Compared with AJCC system, our model displays higher net benefit at a threshold probability around 0.19 or more for 1‐year outcomes and 0.46 or more for 2‐year outcomes, respectively.

Accuracy analysis showed that the NRI for 1‐year prognosis of the new model in the validation set was 0.327 (95% CI: 0.277–0.379), and for 2‐year prognosis was 0.302 (95% CI: 0.220–0.388). Similarly, the IDI for 1‐ and 2‐year prognosis of the new model in the validation set was 0.138 (*P* < 0.001), 0.130 (*P* < 0.001), respectively. In conclusion, the nomogram showed a superior predictive ability when compared with the original AJCC staging model.

## 4 DISCUSSION

As a common solid tumor, NSCLC often presents distant metastasis late in the course of the disease, where over 15% of the patients present with an M1a stage.^3^, ^24^ The AJCC TNM staging system is currently used for prognostic prediction, since accurate prediction of survival can help physicians choose appropriate treatment.^25^ However, given that in addition to tumor stage, various high‐risk factors affect OS, reliable prognostication in M1a NSCLC patients has not been an exact science and is an unmet need. It has been shown that nomograms can provide more accurate and individualized prediction^26^ and can also visualize influencing factors.^27^ Herein, using a large patient cohort from the SEER database, we constructed a novel nomogram to obtain a more accurate prediction of survival for individual M1a patients.

This nomogram encompasses readily accessible as well as impartial baseline clinicopathologic factors including gender, age, histology, N stage, M1a subgroup, grade, surgery of primary site, lymphadenectomy, as well as chemotherapy. Studies have documented various M1a NSCLC prognostic algorithms that use a few baseline variables. Yin et al.^13^ established an M1a prognostic nomogram (C‐index for OS: 0.710; C‐index for cancer‐specific survival: 0.723) by propensity score matching to ease the influence of confounding variables. Based on the M1a NSCLC cohort, in which 5,976 patients had not been subject to surgery and 386 individuals underwent surgery, they found that tumor resection provided better prognosis. However, the metastatic site in M1a NSCLC was not included in their survival nomogram. Tian and colleagues^12^ published a nomogram (C‐index for OS: 0.772) base on MPE or MPCE, in which ipsilateral MPE indicated the better prognosis than other effusion. Our nomogram, which was established using various characteristics and a larger sample size, is the first prognostic nomogram for NSCLC patients of all metastatic sites in M1a staging. This nomogram has functional enhancements when compared to previously prognostic M1a models.

Tumor grade is recognized as an important prognostic marker and, interestingly, was not incorporated into this risk model. A possible reason is that tumor grade might be correlated with various factors in our model and that these factors could be very efficient. Another reason might be that our model was established with a focus on a specific subgroup, which is M1a patients. Due to their heterogeneity, metastatic tumor cells are more aggressive, which makes primary tumor grades to be less important in prognostic predictions.^12^

Whether surgery is necessary for patients with M1a stage NSCLC remains controversial.^28^, ^29^ Theoretically, surgery can completely remove the tumor foci, reduce the tumor burden and alleviate tumor‐caused complications compared with radiotherapy and chemotherapy. With survival analyses of treatments based on our data, we found that primary site surgery would indicate better OS compared with non‐surgery. It has been well documented that surgery of primary lesions in M1a NSCLC patients can remarkably enhance prognosis.^13^, ^14^ Shen et al.^30^ similarly reported that compared with the M1b stage, patients with an M1a stage had remarkably improved outcomes undergoing surgery.

Chemotherapy, as an important part of multimodality therapy, is vital for the prognostic prediction of M1a NSCLC patients. It is also evident from our nomogram that chemotherapy is involved in the prognosis of M1a NSCLC patients. Liu et al. reported that patients subjected to chemotherapy combined with surgery had a significantly better prognosis than those who received chemotherapy alone or chemoradiotherapy combined with surgery.^31^ Due to the limited information available in the SEER database, targeted therapy was not incorporated into this study. Targeted therapies have been shown to provide significant clinical benefit in patients with advanced lung cancer.^32^, ^33^ A meta‐analysis by Liu and colleagues^34^ found that targeted therapy in combination with chemotherapy prolonged the progression‐free survival (PFS) compared with chemotherapy alone (hazard ratio, 0.82; 95% CI: 0.78–0.87). Radiotherapy, as another important modality for tumor treatment, was not included during the variable screening process, indicating that radiotherapy has a minimal prognostic impact in M1a NSCLC patients. However, as a method for palliative treatment, radiotherapy can play a role in alleviating patient suffering and controlling tumor progression.^35^

Among all the pathological types of NSCLC patients, ADC has the best prognosis, which was the same as in previous studies.^2^, ^11^, ^12^ This may be because ADC exhibits extra EGFR gene mutations, which makes ADC to be more sensitive to EGFR‐TKIs, so that patients can benefit from anti‐EGFR regimens. Among the M1a descriptors, MPE or MPCE suggested a poorer prognosis. Previous study also found that patients with MPE or MPCE had poor survival outcomes.^6^ Therefore, whether to subclassify this heterogeneous patient population still needs to be considered. Overall, our nomogram contains reasonable factors that can effectively predict the prognosis of different M1a NSCLC patients. The established nomogram is a more precise prognostic model when compared to the TNM staging system.

It is important to point out that this study has several limitations. First, this is a retrospective study, some clinicopathological variable including body mass index, smoking status, serum markers, the usage of EGFR‐TKIs, detailed regimens of chemotherapy,^34^, ^36^ as well as molecular markers, that might improve model accuracy, were not included in the current study since these data are not provided by the SEER database. Second, only the prognosis of a single metastatic site, that is*,* M1a subgroup, was analyzed, and no information was provided for cases with a subgroup combination, such as “STCL + PTIL”, which leads to some limitations in our nomogram in the clinical assessment of patient prognosis. Third, our results showed that PTIL had a better prognosis than STCL and MPE/MPCE, but the number as well as the location of pleural nodules were not recorded in the SEER database in detail, which may have implications for the analysis. Fourth, all patients in this study were grouped according to the seventh edition of the AJCC‐TNM staging system, however, coding rules on tumor extension made it difficult to restage the patients based on the latest eighth edition of the TNM classification.^11^ In addition, our nomogram is only based on data for USA patients, and thus, is not representative of global patients. Therefore, studies using global prospective data, the latest TNM classification system as well as comprehensive prognostic factors should be performed to improve our model.

## 5 CONCLUSION

Our study established a nomogram for the prediction of 1‐ and 2‐year OS in patients with NSCLC diagnosed with stage M1a, facilitating clinical workers to accurately evaluate the individual survival of M1a NSCLC patients. The accuracy and clinical applicability of this nomogram were validated.

## CONFLICT OF INTEREST

The authors have no conflict of interest.

## AUTHOR CONTRIBUTIONS

Hongchao Chen: Study concept and design, writing (original draft), data collection, analyses, and interpretation. Chen Huang: Funding for publication, writing (review and editing), data collection, analyses, and interpretation. Huiqing Ge: Data collection, analyses, and interpretation. Qianshun Chen: Data collection, analyses, and interpretation. Jing Chen: Data collection, analyses, and interpretation. Yuqiang Li: Data collection, analyses, and interpretation. Haiyong Chen: Data collection, analyses, and interpretation. Shiyin Luo: Data collection, analyses, and interpretation. Lilan Zhao: Study concept and design, writing (review and editing), study supervision, study concept and design, and project administration. Xunyu Xu: Funding acquisition, study supervision, study concept and design, and project administration. All authors read and approved the final manuscript.

## ETHICS APPROVAL STATEMENT

We signed the “Surveillance, Epidemiology, and End Results Program Data‐Use Agreement” in accordance with the requirement of using SEER database.

## Data Availability

The datasets analyzed during the current study are available in the SEER repository (https://seer.cancer.gov/). The source code was released on GitHub (https://seer.cancer.gov/).
